# Protease-Activated Receptor-1 Antagonist Protects Against Lung Ischemia/Reperfusion Injury

**DOI:** 10.3389/fphar.2021.752507

**Published:** 2021-09-30

**Authors:** Shi-Jye Chu, Shih-En Tang, Hsin-Ping Pao, Shu-Yu Wu, Wen-I Liao

**Affiliations:** ^1^ Department of Internal Medicine, National Defense Medical Center, Tri-Service General Hospital, Taipei, Taiwan; ^2^ Division of Pulmonary and Critical Care, Department of Internal Medicine, National Defense Medical Center, Tri-Service General Hospital, Taipei, Taiwan; ^3^ Institute of Aerospace and Undersea Medicine, National Defense Medical Center, Taipei, Taiwan; ^4^ The Graduate Institute of Medical Sciences, National Defense Medical Center, Taipei, Taiwan; ^5^ Department of Emergency Medicine, National Defense Medical Center, Tri-Service General Hospital, Taipei, Taiwan

**Keywords:** acute lung injury, ischemia-reperfusion, protease-activated receptor 1, thrombin, SCH530348

## Abstract

Protease-activated receptor (PAR)-1 is a thrombin-activated receptor that plays an essential role in ischemia/reperfusion (IR)-induced acute inflammation. PAR-1 antagonists have been shown to alleviate injuries in various IR models. However, the effect of PAR-1 antagonists on IR-induced acute lung injury (ALI) has not yet been elucidated. This study aimed to investigate whether PAR-1 inhibition could attenuate lung IR injury. Lung IR was induced in an isolated perfused rat lung model. Male rats were treated with the specific PAR-1 antagonist SCH530348 (vorapaxar) or vehicle, followed by ischemia for 40 min and reperfusion for 60 min. To examine the role of PAR-1 and the mechanism of SCH530348 in lung IR injury, western blotting and immunohistochemical analysis of lung tissue were performed. *In vitro*, mouse lung epithelial cells (MLE-12) were treated with SCH530348 or vehicle and subjected to hypoxia-reoxygenation (HR). We found that SCH530348 decreased lung edema and neutrophil infiltration, attenuated thrombin production, reduced inflammatory factors, including cytokine-induced neutrophil chemoattractant-1, interleukin-6 and tumor necrosis factor-α, mitigated lung cell apoptosis, and downregulated the phosphoinositide 3-kinase (PI3K), nuclear factor-κB (NF-κB) and mitogen-activated protein kinase (MAPK) pathways in IR-injured lungs. In addition, SCH530348 prevented HR-induced NF-κB activation and inflammatory chemokine production in MLE12 cells. Our results demonstrate that SCH530348 exerts protective effects by blocking PAR-1 expression and modulating the downstream PI3K, NF-κB and MAPK pathways. These findings indicate that the PAR-1 antagonist protects against IR-induced ALI and is a potential therapeutic candidate for lung protection following IR injury.

## Introduction

Acute lung injury (ALI) and acute respiratory distress syndrome (ARDS) are fatal illnesses marked by diffuse lung inflammation, disruption of the alveolar barrier, high vascular permeability, and subsequent pulmonary edema. ALI is a complex process characterized by marked neutrophil, T cell and macrophage infiltration in the interstitial and alveolar spaces (Nadeem et al., 2020). Numerous inflammatory and oxidative mediators are involved in the development of ALI. Increased expression of proinflammatory cytokines released from infiltrated neutrophils and T cells, including interleukin (IL)-1β, IL-6, IL-17A and tumor necrosis factor-α (TNF-α), further exacerbates pulmonary inflammation (Ahmad et al., 2019; Nadeem et al., 2019; Ahmad et al., 2020). Excessive production of reactive oxygen species (ROS) by nicotinamide adenine dinucleotide phosphate oxidase activation in neutrophils is the other mechanism mediating ALI (Nadeem et al., 2019). Multiple risk factors contribute to ALI, one of which is ischemia/reperfusion (IR) injury. During ischemia, hypoxia induces the generation of ROS and proinflammatory cytokines and activates survival pathways, including nuclear factor-κB (NF-κB) and mitogen-activated protein kinase (MAPK) signaling (Eltzschig and Eckle, 2011). Upon reperfusion of the ischemic lung, uncontrolled activation of thrombin induces thromboinflammatory responses and promotes neutrophil recruitment and extravasation into the postischemic tissues, further amplifying IR-induced inflammatory injury (Jackson et al., 2019). To date, there is no effective pharmacotherapy to decrease IR-induced ALI-associated mortality, highlighting the need for novel therapeutic strategies.

Mounting evidence has shown that widespread microvascular thrombosis increases the inflammatory response, endothelial cell permeability and pulmonary fluid leakage in sepsis and IR injury (Jackson et al., 2019; Li et al., 2020). Thrombin, a multipotent serine protease, is generated during coagulation cascade activation and triggers proinflammatory and proapoptotic signaling via the activation of protease-activated receptor (PAR)-1 (Claushuis et al., 2017). Thrombin inhibition decreases thrombin-antithrombin complex (TAT) levels in bronchoalveolar lavage fluid (BALF) and ameliorates lung injury in rats with pneumococcal pneumonia infection (Choi et al., 2008). Furthermore, antithrombin therapy has shown an anti-inflammatory effect and could attenuate brain damage after cerebral ischemia in mice (Nakano et al., 2020).

PARs are G protein-coupled receptors that are widely expressed in platelets, endothelial cells, epithelial cells and smooth muscle cells and mediate the cellular response to specific proteases. Among the four identified types of PARs (PAR-1 to PAR-4), PAR-1 is essential for the regulation of endothelial barrier function and proinflammatory factor release and mediates the interplay between coagulation and inflammation in various inflammatory conditions, including sepsis, multiple sclerosis, and ALI (Chambers, 2008; Chambers and Scotton, 2012; Kim et al., 2015; Li et al., 2020). Thrombin irreversibly activates PAR-1 by cleaving the amino terminus and exposes a tethered peptide ligand that then transactivates the receptor during thromboinflammation (Zhang et al., 2012). PAR-1 activation increases microvascular permeability, and PAR-1 antagonists maintain microvascular integrity in rats undergoing subarachnoid hemorrhage (Yan et al., 2013). The activation of PAR-1 on epithelial cells can also induce the release of granulocyte chemotaxis and proinflammatory mediators (Chambers, 2008). In addition, a PAR-1 antagonist can effectively reduce neutrophil recruitment in LPS- and *S. pneumoniae*-induced lung injury (Howell et al., 2005; Mercer et al., 2014; Jose et al., 2015). Furthermore, siRNA knockdown of PAR-1 suppresses organic dust-induced inflammatory gene induction in lung epithelial cells (Natarajan et al., 2016).

A previous study showed that PAR-1-knockout mice exhibited reduced cerebral infarction volumes and decreased endothelial barrier leakage in the context of cerebral ischemic injury (Rajput et al., 2014). Several studies in various IR models, such as the kidney (El Eter and Aldrees, 2012), liver (Noguchi et al., 2020), heart (Strande et al., 2007), and brain (Wang et al., 2012), have demonstrated that PAR-1 antagonists have protective effects against IR injury. However, the role of PAR-1 antagonists in IR-induced ALI remains unknown. We hypothesized that the inhibition of thrombin-PAR-1 signaling in the pulmonary system attenuates IR-induced ALI. Therefore, we used SCH530348 (vorapaxar), a highly specific, virtually irreversible PAR-1 antagonist (Zhang et al., 2012), in an isolated IR lung model to investigate this hypothesis.

## Materials and Methods

### Animals

The Institutional Animal Care and Use Committee of the National Defense Medical Center approved all procedures involving rats. Sprague-Dawley rats (male, 350 ± 20 g) were housed in individual cages with free access to food and water in accordance with the National Institutes of Health guidelines (National Academy Press, 1996).

### Isolated Perfused Lung Model in Rats

In brief, each rat was ventilated with humidified air containing 5% CO_2_ and a 1-cm H_2_O positive end-expiratory pressure via tracheostomy. Mechanical ventilation was implemented with a tidal volume of 3 ml and 60 breaths/min. Following a median sternotomy, 1 U of heparin/g body weight (BW) was injected into the right ventricle, and 10 ml of blood was aspirated via cardiac puncture. A cannula was placed into the pulmonary artery, and another wide-bore cannula was inserted into the left ventricle. The pulmonary venous pressure and pulmonary arterial pressure were continuously recorded from the side arm of the cannula. The isolated lung was perfused in 10 ml of previously collected blood and 10 ml of physiological salt solution (PSS) containing 4% bovine serum albumin. The PSS included 50 mM sucrose, 5.5 mM glucose, 1.17 mM MgSO_4_, 1.18 mM KH_2_PO_4_, 22.6 mM NaHCO_3_, 1.6 mM CaCl_2_, 4.7 mM KCl, and 119 mM NaCl. A roller pump was used to maintain a constant circuit flow rate of 8 ml/min. An electronic balance was placed under the isolated perfused lung *in situ,* and real-time lung weight (LW) changes were measured (Chu et al., 2002).

### Experimental Design

The rat lungs were randomly assigned to the control + dimethyl sulfoxide (DMSO, control group), control + SCH530348 (5 mg/kg BW), IR + DMSO (IR group) and IR with different doses of SCH530348 (1, 2.5, or 5 mg/kg BW, *n* = 6 per group). SCH530348 (Adooq Bioscience, Irvine, CA) was dissolved in 0.5% DMSO in saline and injected intraperitoneally 60 min prior to IR. The doses of SCH530348 in this study were chosen based on previous studies (Jose et al., 2015). To induce lung ischemia, the rat lungs were deflated by stopping ventilation and perfusion for 40 min. After this, ventilation was restored, and the lungs were allowed to reperfuse for 60 min in the IR group.

### LW/BW and Wet/Dry Weight Ratios

After the isolated perfused lung experiments, the right middle lung was removed from the hilar region and measured as the wet LW. Then, the wet LW was divided by the BW to determine the LW/BW ratio. The right middle lung was dried at 60°C for 48 h in an oven and then measured to determine the dry weight. The W/D weight ratio was calculated as the wet weight divided by the dry weight.

### Vascular Filtration Coefficient (K_f_)

The K_f_ was determined by venous pressure increase-induced LW change as previously described (Wu et al., 2015). The K_f_ was calculated as the *y*-intercept of the plot (in g·min^−1^) divided by the pulmonary venous pressure (10 cm H_2_O) and LW. The K_f_ is expressed as g·min^−1^ cm H_2_O^−1^ × 100 g.

### Assessment of TAT, Cytokine-Induced Neutrophil Chemoattractant 1 (CINC-1), IL-6, and TNF-*α* Levels in BALF

Coagulation activation was determined by measuring TAT levels in BALF by ELISA (Cloud-Clone Corp., Katy, TX, United States). Cytokines (CINC-1, IL-6, and TNF-α) were quantified with commercial mouse ELISA kits (R&D Systems Inc., Minneapolis, MN, United States) according to the manufacturer’s instructions.

### PAR-1, Myeloperoxidase and Thrombin Immunohistochemical Analysis

Formalin-fixed paraffin lung sections (4 µm) were deparaffinized and pretreated for antigen retrieval, and endogenous peroxidase activity was blocked with 3% H_2_O_2_ in methanol for 15 min. The lung sections were immunostained with rabbit polyclonal antibodies against PAR-1 (1:300, Bioss Antibodies, Woburn, Massachusetts, United States), MPO (1:100, Cell Signaling Technology, Danvers, MA, United States) and thrombin (1:200; LifeSpan Biosciences, Inc., Washington, United States). The lung sections were washed with phosphate-buffered saline twice and then incubated with rat-specific horseradish peroxidase-conjugated secondary antibodies (Nichirei Corporation, Tokyo, Japan) for 30 min. Horseradish peroxidase was visualized after exposure to diaminobenzidine for 3 min. Then, the lung sections were counterstained with hematoxylin.

### Western Blotting

Lung tissue and cultured cell protein lysates (30–50 μg/lane) were separated by 8–12% sodium dodecyl sulfate-polyacrylamide gel electrophoresis, and the proteins were transferred onto polyvinylidene fluoride membranes by electroblotting. Membranes were blocked in 5% milk for 2 h and then incubated overnight at 4°C with one of the following primary antibodies: anti-PAR-1 (1:1,000, Bioss, Woburn, Massachusetts, United States), anti-Bcl-2 (1:500, Bioss), anti-Lamin B1 (1:1,000, Santa Cruz Biotechnology, Dallas, TX, United States), anti-NF-κB p65 (1:800, Cell Signaling Technology), anti-phospho-NF-κB p65 (1:800, Cell Signaling Technology), anti-inhibitor of nuclear factor-κB (IκB)-α (1:800, Cell Signaling Technology), anti-phosphatidylinositol 3-kinase (PI3K) (1:1,000, Cell Signaling Technology), anti-Akt (1:1,000, Cell Signaling Technology), anti-phospho-Akt (1:1,000, Cell Signaling Technology), extracellular signal-related protein kinase 1/2 (ERK1/2) (1:1,000, Cell Signaling Technology), phospho-ERK1/2 (1:1,000, Cell Signaling Technology), anti-c-Jun N-terminal kinase (JNK) (1:1,000, Cell Signaling Technology), anti-phospho-JNK (1:1,000, Cell Signaling Technology), anti-p38 (1:1,000, Cell Signaling Technology), anti-phospho-p38 (1:1,000, Cell Signaling Technology) or β-actin (1:10,000, Sigma Chemical Company, St. Louis, MO, United States). The blots were then washed with phosphate-buffered saline containing 0.1% Tween-20 for 10 min and incubated with rabbit or mouse horseradish peroxidase-conjugated secondary IgG antibodies (1:20,000) for 1 h at room temperature. An enhanced chemiluminescence detection system was used to detect antibody-specific proteins. The data are presented as the relative ratio of the target protein to the reference protein. The relative ratio of the target protein in the control group was arbitrarily expressed as 1.

### Histopathological Analysis

The neutrophil count and lung injury score in the lung tissue were measured as previously described (Wu et al., 2015). Briefly, lung tissues were fixed, sectioned, and stained with hematoxylin and eosin (HE). For all morphological assessments, neutrophil infiltration in the airspace or vessel wall, as well as alveolar capillary interstitial edema, were analyzed in 10 random visual fields at 200× magnification. Furthermore, the degree of lung injury was morphologically assessed by two blinded pathologists using a four-point scale: none (0), mild (1), moderate (2), or severe (3). The resulting two scores were summed to represent the lung injury score (Wu et al., 2015).

### Cell Culture and Induction of Hypoxia-Reoxygenation

Mouse lung epithelial cells (MLE-12 cells) were obtained from ATCC (Manassas, VA, United States) and grown on plastic cell culture dishes in DMEM/F12 K medium containing 4% fetal bovine serum, 0.005 mg/ml insulin, 0.01 mg/ml transferrin, 30 nM sodium selenite, 10 nM hydrocortisone, 10 nM β-estradiol, 10 mM HEPES, and 2 mM l-glutamine. Cells were exposed to 1 or 2 h of hypoxia (1% O_2_-5% CO_2_-94% N_2_) followed by 1 h of reoxygenation (5% CO_2_-95% air). MLE-12 cells were pretreated with 10 μM SCH530348 for 30 min before HR challenge (Abu El-Asrar et al., 2016). The cells were harvested at the indicated time points, and the expression of IκB-α and p-p65 was analyzed by western blotting. β-actin and total p65 were used as loading controls. The cell supernatants were collected and assayed for chemokine (C-X-C motif) ligand 1 (CXCL1) using a mouse CXCL1 ELISA kit (R&D, Inc., Minneapolis, MN, United States).

### Statistical Analysis

GraphPad Prism 5 statistical software (GraphPad Software, San Diego, CA, United States) was used to perform statistical analyses. The data are reported as the mean values ±standard deviation (SD). The data were analyzed using one-way analysis of variance (ANOVA) followed by a post hoc Bonferroni test to compare the differences among groups. Two-way ANOVA with a Bonferroni test was performed to analyze the LW gain between groups. A value of *p* < 0.05 was considered significant.

## Results

### SCH530348 Ameliorated IR-Induced Lung Edema

**FIGURE 1:**
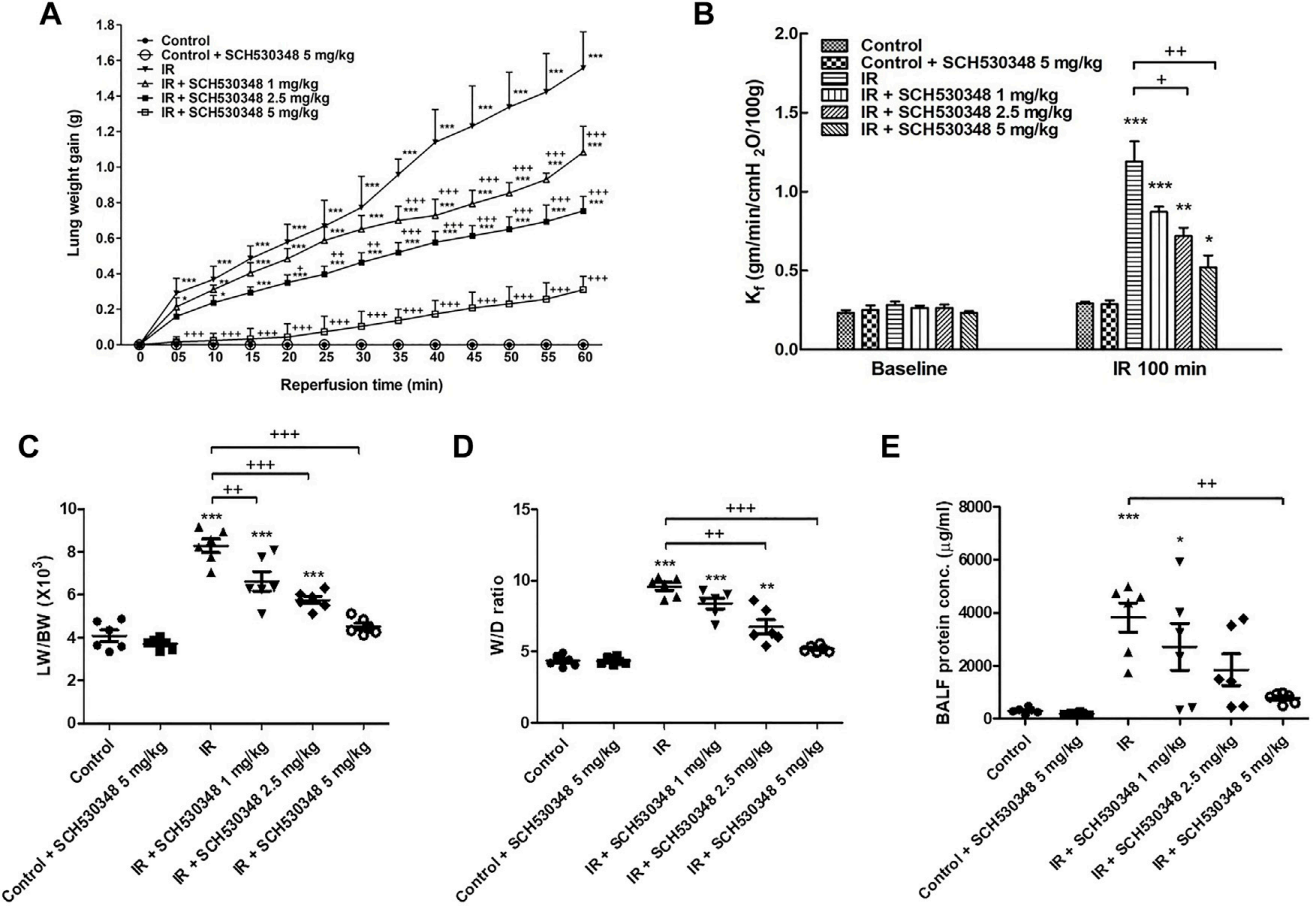
SCH530348 alleviated IR-induced acute lung edema. **(A)** LW gain, **(B)** K_f_, **(C)** LW/BW ratio, **(D)** W/D weight ratio, and **(E)** protein concentration in BALF significantly increased in the IR group. The increases in these parameters were significantly ameliorated by SCH530348. Compared with other doses, 5 mg/kg SCH530348 was more effective in decreasing lung edema. The data are expressed as the mean ± SD (*n* = 6 per group). *p < 0.05, **p < 0.01, ***p < 0.001, compared with the control group; + *p* < 0.05, ++ *p* < 0.01, +++ *p* < 0.001, compared with the IR group.

The LW gain (F = 1,536.8, *p* < 0.001), K_f_ (F = 28.3, *p* < 0.001), LW/BW ratio (F = 41.2, *p* < 0.001), W/D weight ratio (F = 55.7, *p* < 0.001), and protein concentrations in BALF (F = 8.7, *p* < 0.001) were significantly increased by IR injury. Compared with the IR group, the IR + SCH530348 group exhibited dose-dependent reductions in all lung injury parameters (Figure 1).

### SCH530348 Reduced IR-Induced PAR-1 Expression

**FIGURE 2:**
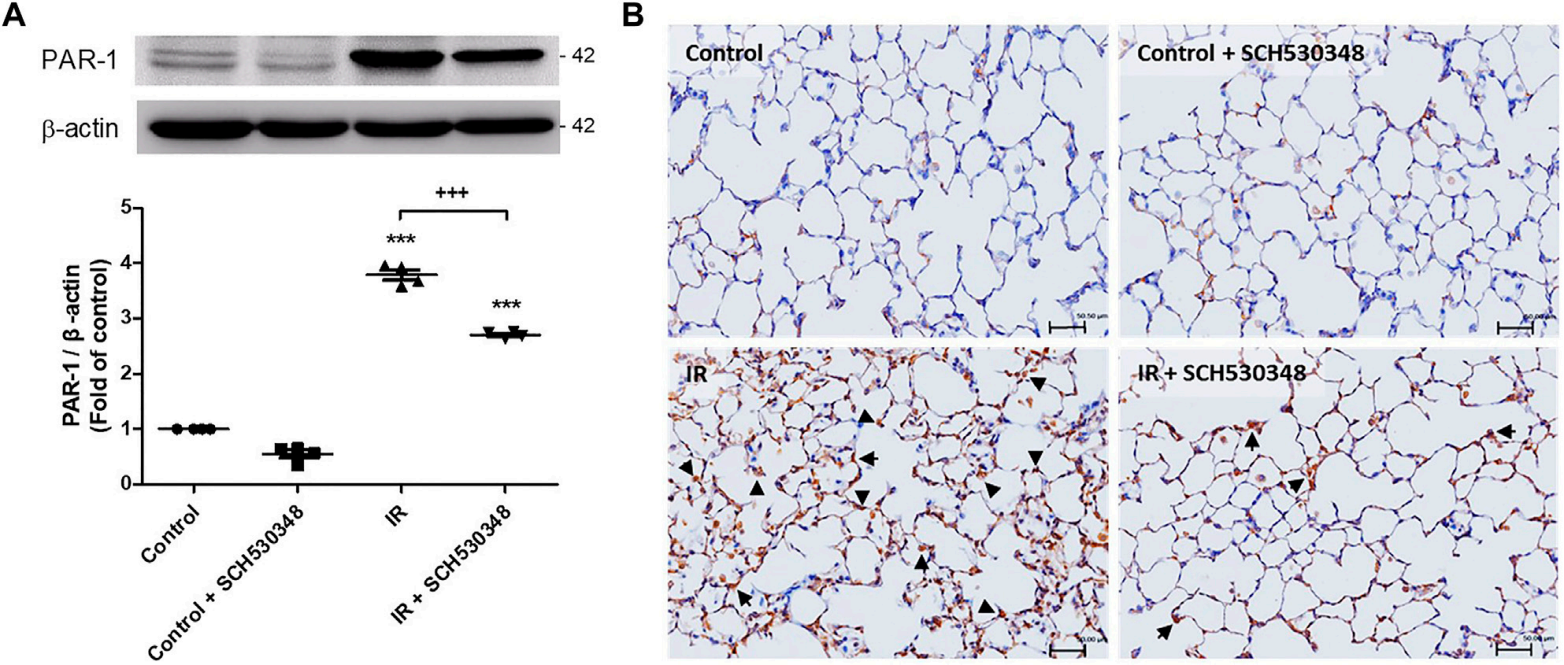
SCH530348 decreased IR-induced PAR-1 expression. **(A)** PAR-1 protein levels were determined by western blotting with specific antibodies. **(B)** IHC analysis of lung slices revealed that PAR-1 expression (indicated by arrowheads) was increased by IR injury compared with that of the control group. SCH530348 treatment decreased PAR-1 expression in the context of IR injury. The data are expressed as the mean ± SD (*n* = 4 per group). ***p < 0.001, compared with the control group; +++ *p* < 0.001, compared with the IR group.

PAR-1 expression was evaluated using western blotting and IHC. IR injury strongly enhanced PAR-1 expression in lung tissue compared with that of the control group (F = 635.8, *p* < 0.001). However, this expression in lung IR injury was significantly reduced by treatment with SCH530348 (Figure 2).

### SCH530348 Decreased IR-Induced Proinflammatory Cytokine Production

**FIGURE 3:**
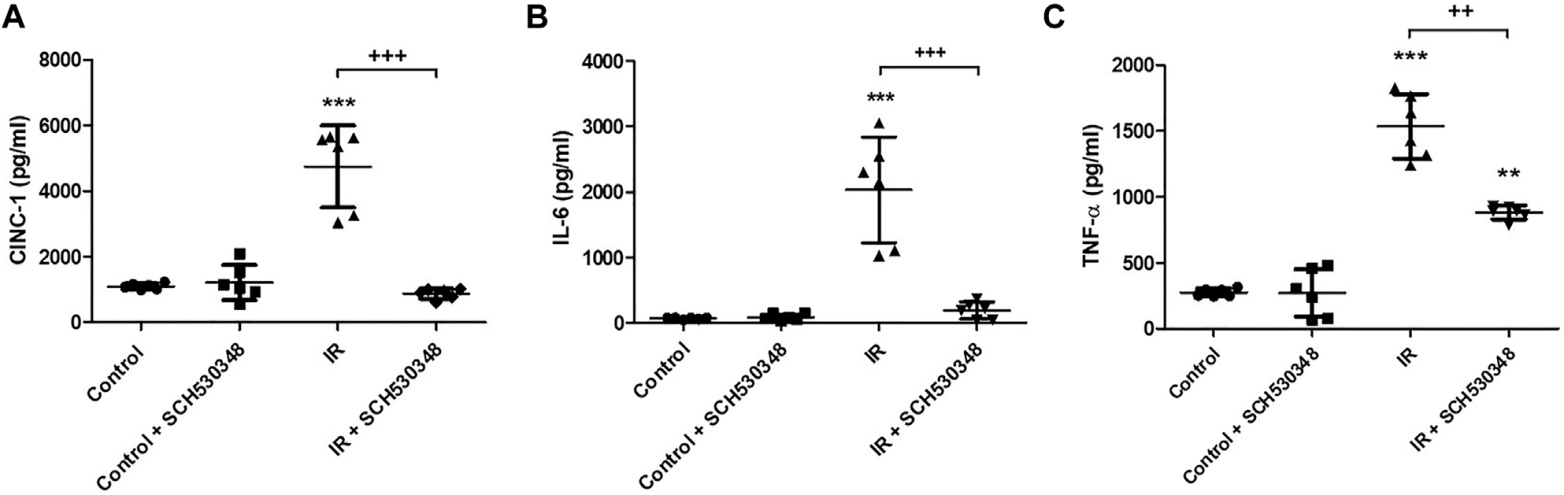
SCH530348 ameliorated IR-induced proinflammatory cytokine production. **(A)** CINC-1, **(B)** IL-6, and **(C)** TNF-α levels in BALF were measured using ELISA kits. CINC-1, IL-6 and TNF-α levels were all significantly elevated in the IR group compared with the control group. SCH530348 treatment significantly attenuated the production of these cytokines in the context of IR injury. The data are expressed as the mean ± SD (*n* = 6 per group). **p < 0.01, ***p < 0.001, compared with the control group; ++ *p* < 0.01, +++ *p* < 0.001, compared with the IR group.

To characterize the inflammatory mediator response to PAR-1 signaling in IR injury, we quantitatively analyzed several proinflammatory mediators, including CINC-1, IL-6, and TNF-α, in BALF following SCH530348 treatment. The results showed that IR could significantly increase the production of CINC-1 (F = 43.5, *p* < 0.001), IL-6 (F = 32.8, *p* < 0.001), and TNF-α (F = 91.8, *p* < 0.001) in BALF. Compared with the IR group, the IR + SCH530348 group exhibited markedly decreased cytokine levels after IR injury. These results demonstrate that SCH530348 inhibited proinflammatory cytokine production in lung IR injury (Figure 3).

### SCH530348 Improved IR-Induced Lung Histopathological Change

**FIGURE 4:**
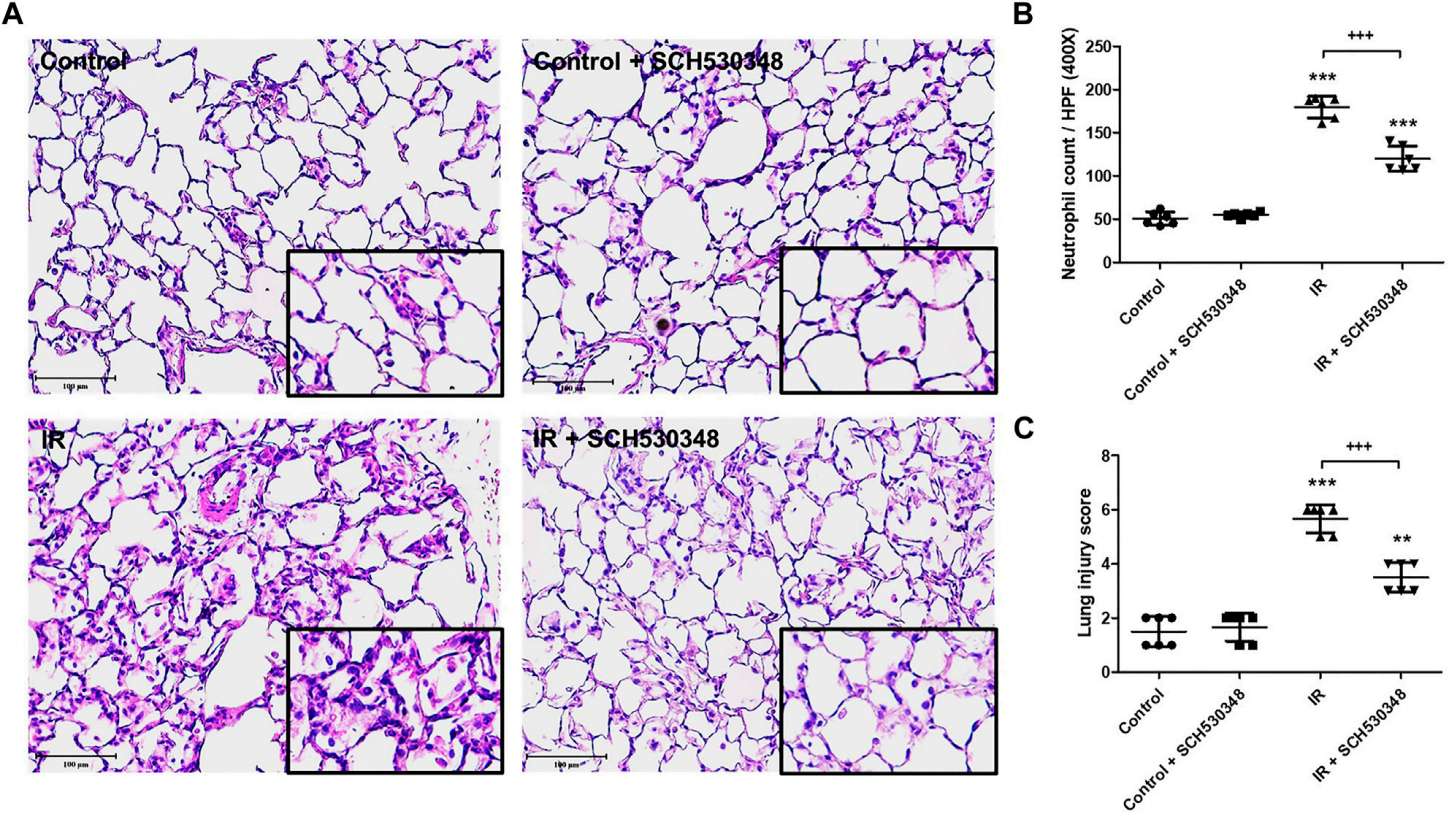
SCH530348 mitigated IR-induced acute lung injury. **(A)** HE staining of lung tissue showed thickening of alveolar septa and neutrophil infiltration in the interstitial and alveolar spaces after IR injury compared with that of the sham control. Alveolar damage and neutrophil recruitment after IR injury were attenuated by SCH530348 treatment. **(B)** The number of infiltrated neutrophils per high-power field (400x magnification) was elevated in the IR group, while additional SCH530348 significantly reduced the number of neutrophils in the context of IR injury. **(C)** SCH530348 significantly decreased IR-induced elevations in lung injury scores. Cell counting was performed at 400 x magnification; scale bar = 100 μm. The data are expressed as the mean ± SD (*n* = 6 per group). **p < 0.01, ***p < 0.001, compared with the control group; +++ *p* < 0.001, compared with the IR group.

Representative images of HE-stained lung sections from the IR or control group are shown in Figure 4. Histopathological analysis of the lung showed increases in neutrophil infiltration and alveolar wall thickness in the IR group. However, these changes were markedly attenuated in SCH530348-treated rats compared with vehicle-treated rats after IR injury (Figure 4A). SCH530348 treatment significantly decreased neutrophil counts (F = 207.9, *p* < 0.001) in the lungs and histological lung injury scores (F = 80.2, *p* < 0.001) in the context of IR injury (Figures 4B,C).

### SCH530348 Suppressed IR-Induced MPO Activity

**FIGURE 5:**
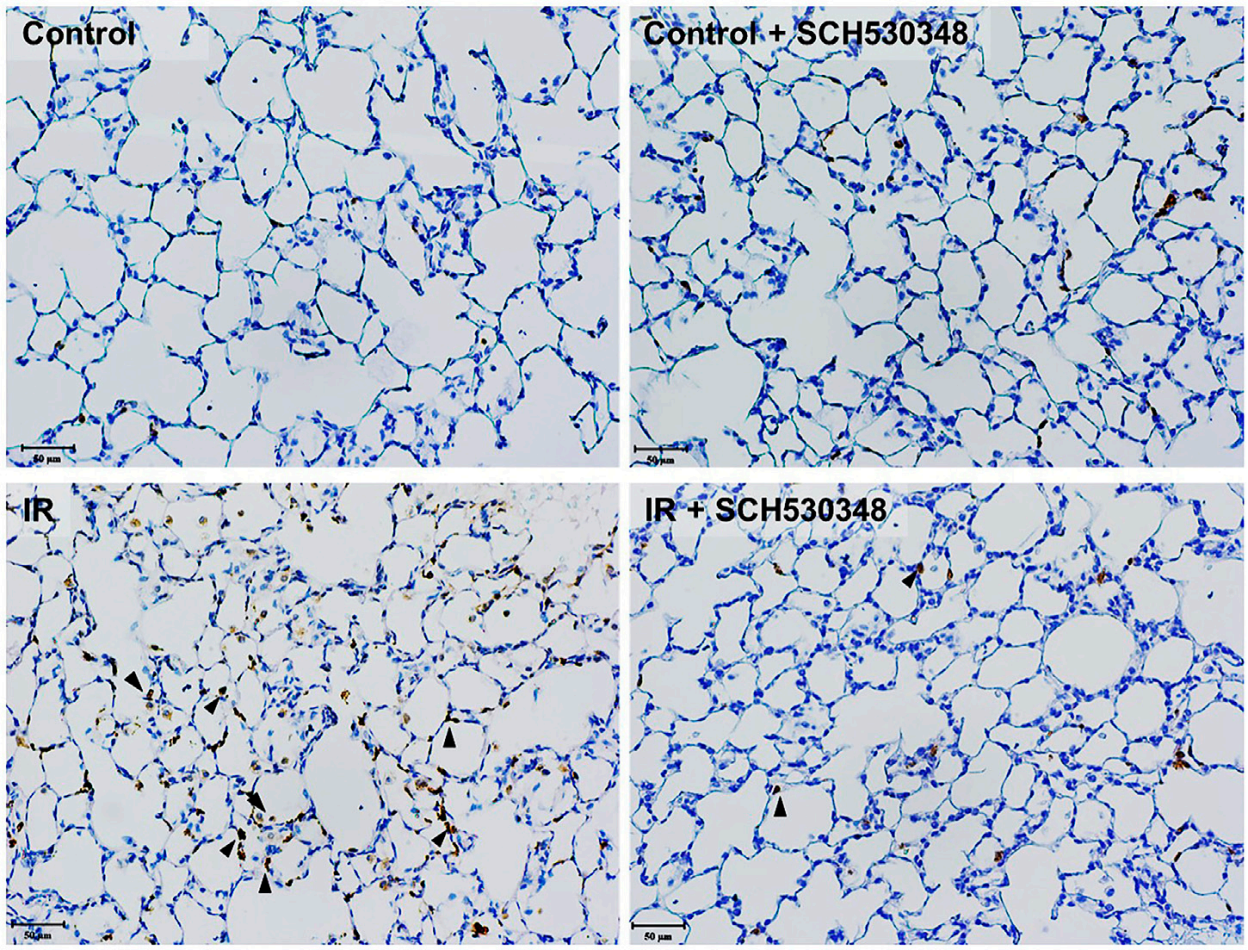
SCH530348 decreased IR-induced MPO activity. Serial paraffin-embedded sections from IR rats were stained for the neutrophil marker MPO. MPO-positive cells are indicated by arrowheads (200× magnification). The number of MPO-positive cells in lung tissue was increased following IR injury, and SCH530348 treatment markedly attenuated this increase.

Lung MPO activity, a marker of neutrophil accumulation, was analyzed by IHC. Figure 5 shows that IR injury induced marked MPO-positive cells in the lung sections compared with those of the control group. Rats treated with SCH530348 had fewer MPO-positive cells than vehicle-treated rats after IR injury.

### SCH530348 Attenuated IR-Induced Thrombin Activity in BALF

**FIGURE 6:**
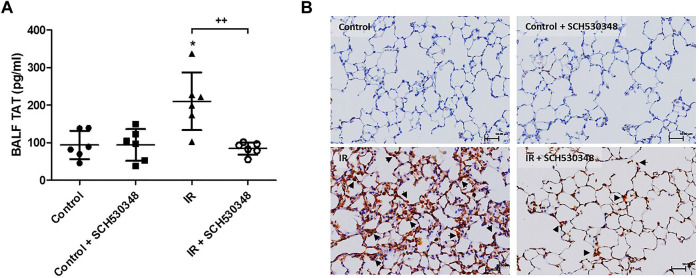
SCH530348 downregulated IR-induced thrombin activity. **(A)** TAT levels in BALF were measured by ELISA. **(B)** Representative images showing IHC staining of thrombin in lung sections from the vehicle or IR group (200× magnification). Scale bars, 50 μm. TAT levels in BALF and thrombin-positive cells in lung tissue (indicated by arrowheads) were increased in the IR group compared with the control group. SCH530348 treatment mitigated these increases. The data are expressed as the mean ± SD (*n* = 6 per group). *p < 0.05, compared with the control group; ++ *p* < 0.01, compared with the IR group.

In rats with IR injury, marked increases in BALF TAT levels were observed compared with those in vehicle-treated rats. IR-induced increases in TAT levels were attenuated by SCH530348 treatment (F = 9.1, *p* < 0.01; Figure 6A). Thrombin-positive cells were increased in the interstitial and alveolar spaces after IR injury compared with those of the control group, and SCH530348 treatment reversed this effect (Figure 6B).

### SCH530348 Reduced Apoptosis in the Lung After IR Injury

**FIGURE 7:**
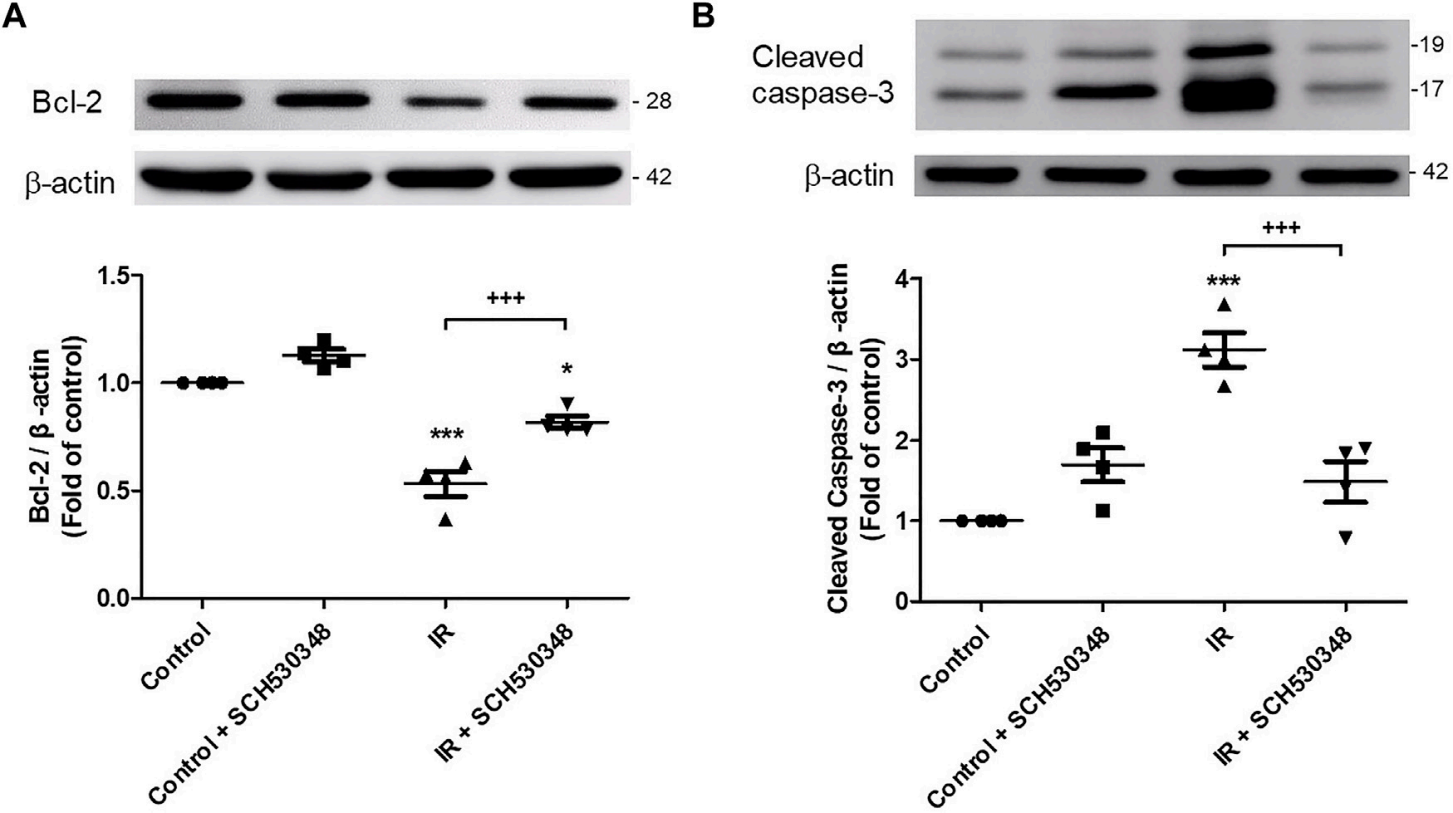
SCH530348 reduced IR-induced lung apoptosis. Western blot analysis of **(A)** Bcl-2 and **(B)** cleaved caspase-3 protein expression in lung tissue. β-actin served as a loading control. Representative blots are shown. IR significantly decreased Bcl-2 and induced cleaved caspase-3 protein expression in lung tissue. SCH530348 treatment significantly reversed these IR-induced apoptotic effects. The data are expressed as the mean ± SD (*n* = 4 per group). *p < 0.05, ***p < 0.001, compared with the control group; +++ *p* < 0.001, compared with the IR group.

Bcl-2 and cleaved caspase-3 activity were analyzed by western blotting. IR injury significantly decreased Bcl-2 (F = 54.9, *p* < 0.001) and increased cleaved caspase-3 protein (F = 21.7, *p* < 0.001) expression compared with that of the control group. SCH530348 significantly reversed these IR-induced apoptotic effects (Figure 7).

### SCH530348 Attenuated IR-Induced PI3K and NF-κB Signaling Activation

**FIGURE 8:**
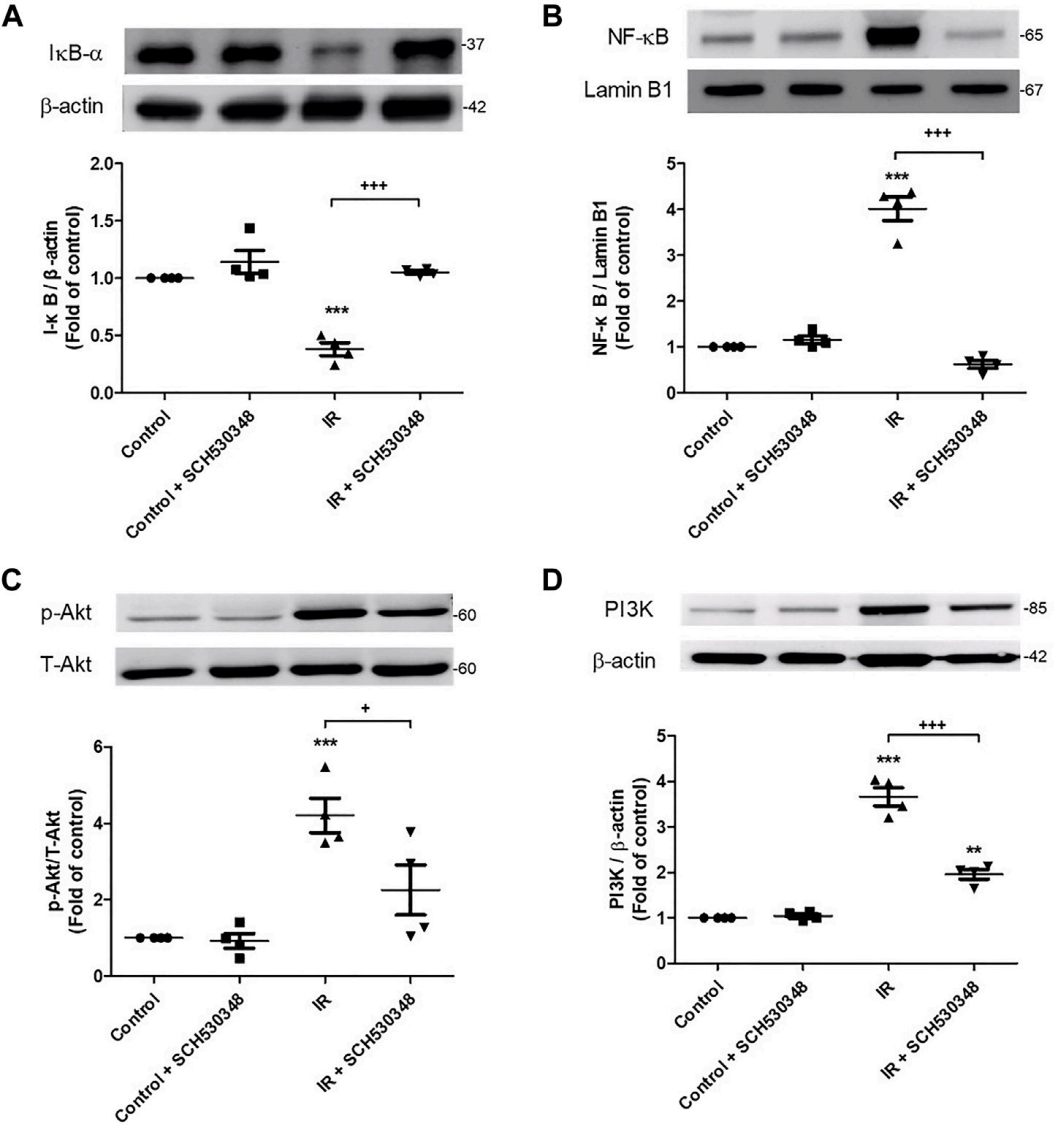
SCH530348 suppressed IR-induced activation of PIK3 and NF-κB signaling. The protein levels of **(A)** IκB, **(B)** NF-κB, **(C)** Akt and **(D)** PI3K were detected by western blotting. Lamin B1 and β-actin served as loading controls for nuclear and cytoplasmic proteins, respectively. The protein levels of NF-κB, Akt and PI3K in lung tissue significantly increased and the protein level of IκB significantly decreased following exposure to IR. In addition, SCH530348 significantly attenuated the nuclear translocation of NF-κB, the degradation of IκB, and Akt and PI3K protein expression in the context of IR injury. The data are expressed as the mean ± SD (*n* = 4 per group). **p < 0.01, ***p < 0.001, compared with the control group; + *p* < 0.05, +++ *p* < 0.001, compared with the IR group.

To identify the downstream effectors involved in the effects of SCH530348, we assessed PI3K, Akt, NF-κB and IκB-α protein expression by western blotting. PI3K (F = 113.9, *p* < 0.001), Akt (F = 13.9, *p* < 0.001) phosphorylation, and nuclear NF-κB (F = 119.1, *p* < 0.001) were significantly increased and cytosolic IκB-α (F = 36.1, *p* < 0.001) was significantly decreased after lung IR injury, and SCH530348 treatment significantly attenuated these effects (Figure 8).

### SCH530348 Mitigated IR-Induced Activation of MAPK Signaling

**FIGURE 9:**
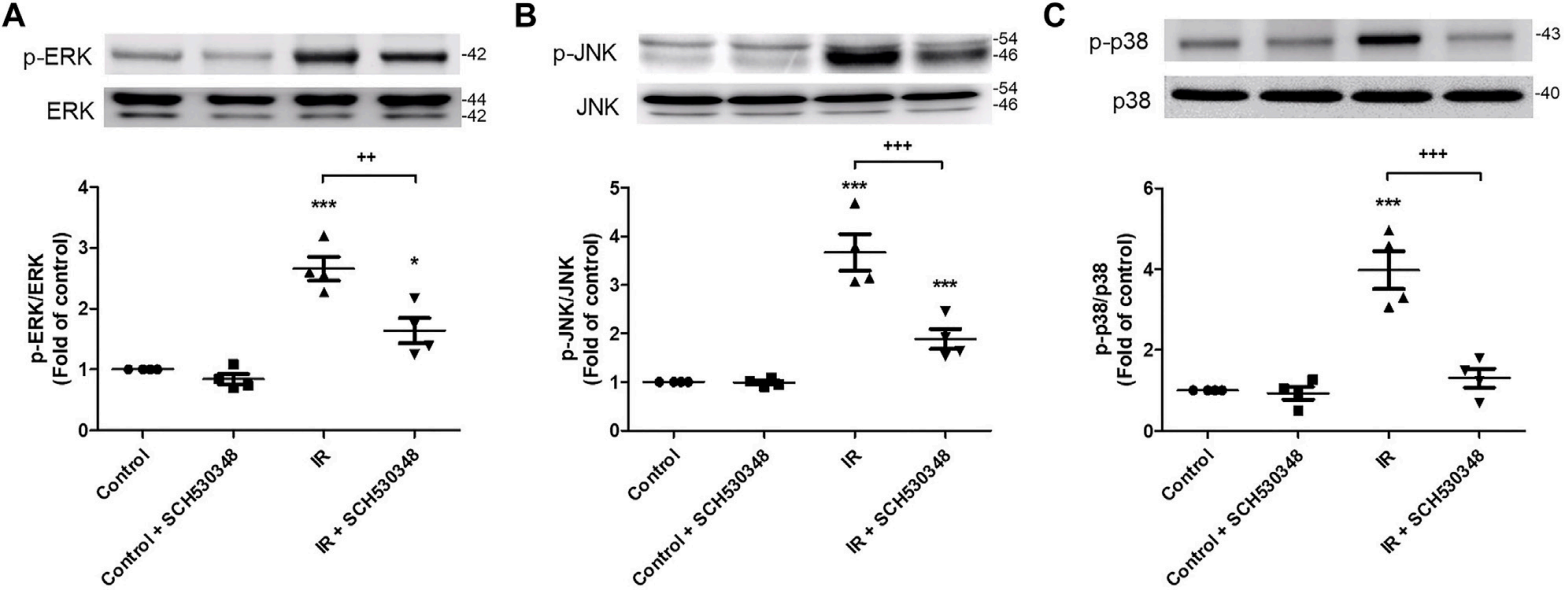
SCH530348 attenuated IR-induced MAPK signaling activation. Western blot analysis showing that the levels of **(A)** phosphorylated ERK, **(B)** JNK and **(C)** p38 were upregulated in lung tissue after IR injury compared with those of the control group. The levels of phosphorylated ERK, JNK and p38 were downregulated after SCH530348 treatment following IR injury. The data are expressed as the mean ± SD (*n* = 4 per group). *p < 0.05, ***p < 0.001, compared with the control group; ++ *p* < 0.01, +++ *p* < 0.001, compared with the IR group.

The effects of SCH530348 on ERK, JNK and p38 protein expression were examined using a western blot analysis. Compared with that of the control group, ERK (F = 30.8, *p* < 0.001), p38 (F = 28.5, *p* < 0.001) and JNK (F = 34.4, *p* < 0.001) phosphorylation was significantly increased following IR injury. However, these effects were significantly attenuated by the administration of SCH530348 (Figure 9).

### SCH530348 Ameliorated HR-Induced NF-κB Activation and CXCL1/KC Production in MLE-12 Cells

**FIGURE 10:**
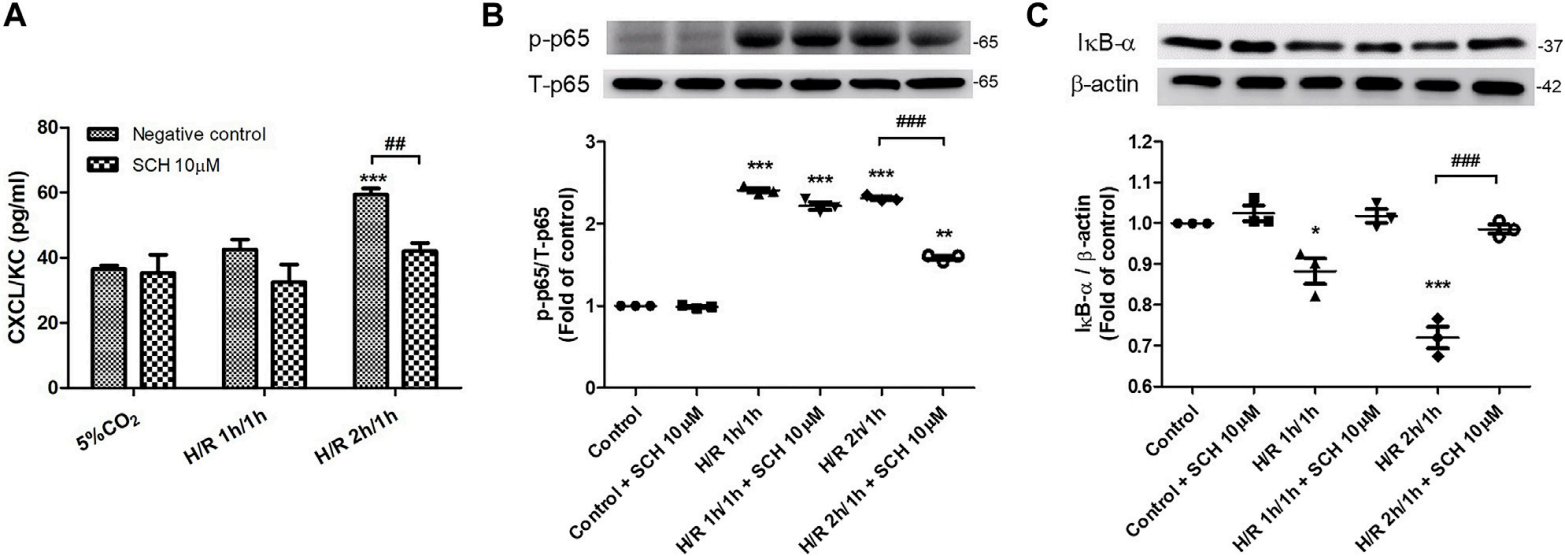
SCH530348 attenuated HR-induced chemokine production and NF-κB signaling activation in MLE-12 cells. Mouse lung epithelial cells were subjected to HR for 3 h and were treated with vehicle or 10 µM SCH530348. **(A)** HR-induced MLE-12 cells produced higher CXCL1/KC levels than control group cells, and SCH530348 significantly decreased chemokine production following HR for 3 h **(B,C)** SCH530348 significantly reduced HR-induced NF-κB p65 phosphorylation and IκB-α degradation. β-actin was used as an internal control. The data are expressed as the mean ± SD (*n* = 3 per group). *p < 0.05, **p < 0.01, ***p < 0.001 compared with the control group; ##*p* < 0.01, ###*p* < 0.001, compared with the HR group.

We observed increased levels of CXCL1/KC and NF-κB phosphorylation and decreased expression of IκB-α in MLE-12 cells subjected to 3 h of HR. Compared with those in the HR group at 3 h, coincubation with 10 μM SCH530348 significantly decreased the levels of CXCL1/KC (F = 42.3, *p* < 0.001) and NF-κB (F = 633.1, *p* < 0.001) phosphorylation and enhanced the expression of IκB-α (F = 34.6, *p* < 0.001; Figure 10).

## Discussion

SCH530348 (vorapaxar) has been approved by the Food and Drug Administration for the prevention of thrombotic cardiovascular events in patients with a history of myocardial infarction. The present study was designed to determine the effect of SCH530348 on IR-induced ALI. Herein, we found that SCH530348 attenuated lung thromboinflammation and the development of ALI after IR injury. Specifically, SCH530348 administration diminished lung PAR-1 activity, reduced thrombin expression in BALF and lung tissue, ameliorated apoptosis, and suppressed PI3K, NF-κB and MAPK signaling activation, thereby reducing proinflammatory cytokine production and subsequent ALI. We also present data regarding the ability of SCH530348 to regulate the activation of NF-κB and decrease proinflammatory chemokine production in MLE-12 cells. In brief, we report a previously unknown role of SCH530348, a PAR-1 antagonist, as a therapeutic candidate for lung IR injury.

Excessive neutrophilic inflammation is a key contributor to alveolar damage in IR-induced ALI. Thrombin-mediated PAR-1 activation is essential for initiating neutrophil recruitment and promoting alveolar-capillary barrier disruption in acute lung inflammation (Chen et al., 2008; Mercer et al., 2014). Furthermore, activation of PAR-1 triggers IL-6 and CXCL1/KC release and increases lung inflammation in mice after influenza infection (Khoufache et al., 2013). Jose et al. (2015) demonstrated that a PAR-1 antagonist decreased inflammatory mediator production and prevented neutrophil recruitment in relation to *S. pneumoniae* pulmonary infection. Recent studies have shown that IR injury upregulates the expression of PAR-1 in the kidney, brain and liver (Xie et al., 2015; Noguchi et al., 2020; Guan et al., 2021). Moreover, Xie et al. (2015) found that blood-brain barrier permeability was highly correlated with PAR-1 expression in cerebral IR injury in rats. Consistent with previous studies, we found that SCH530348 decreased PAR-1 expression, TNF-α, IL-6, and CINC-1 production, neutrophil infiltration, and alveolar damage in lung IR injury. These findings suggest that SCH530348 can attenuate lung IR injury by suppressing PAR-1 signaling and neutrophilic inflammation.

Thrombin is known to be the major activator of PAR-1 and is one of the hallmarks of thromboinflammatory activation. High concentrations of thrombin increase endothelial cell permeability and are detrimental to blood-brain barrier integrity by activating PAR-1 (Bae et al., 2009; Festoff et al., 2016). Thrombin inhibition was previously reported to protect against cerebral IR injury by decreasing proinflammatory cytokine levels (Nakano et al., 2020). In the current study, we found that both TAT levels in BALF and thrombin expression in lung tissue were increased after IR injury, suggesting that thrombin could be an important factor in causing lung IR injury. Moreover, the findings of the current study are consistent with those of Kim, which revealed that PAR-1 antagonist treatment reduced thrombin expression in an experimental autoimmune encephalomyelitis model (Kim et al., 2015). These results confirm that thrombin expression is critical to IR-induced lung injury and that SCH530348 can ameliorate thrombin-mediated thromboinflammation.

Apoptosis is the main mechanism of cell death after lung IR injury (Den Hengst et al., 2010). Lung ischemia generates ROS and proinflammatory cytokines and then triggers the apoptosis pathway, which is regulated mainly by Bcl-2 family proteins (Liao et al., 2021). The cell death pathway is characterized by activation of the caspase cascade, and the cleavage of caspase-3 is a key regulator of apoptosis after reperfusion (Wang et al., 2020; Liao et al., 2021). Previous studies showed that caspase-3 activity was significantly increased and Bcl-2 expression was significantly decreased after IR-induced lung injury (Hung et al., 2019). Thrombin induced apoptosis in human alveolar epithelial cells through PAR-1-dependent modulation of JNK and Akt (Suzuki et al., 2005). A PAR-1 antagonist attenuated apoptosis by inhibiting JNK signaling in rats with surgical brain injury (Manaenko et al., 2013). Furthermore, Noguchi et al. (2020) demonstrated that SCH530348 significantly attenuated hepatic IR injury through an antiapoptotic effect. In our study, SCH530348 increased Bcl-2 and decreased cleaved caspase-3 expression after IR injury, suggesting that SCH530348 ameliorates lung IR injury by blocking the apoptotic cascade.

The present study also explored the protective mechanisms of SCH530348 by assessing signaling pathways downstream of PAR-1, including the PI3K, NF-κB and MAPK pathways. PI3K activation is required for Akt phosphorylation, which plays a crucial role in thromboinflammation and ROS production (Li et al., 2015; Koundouros and Poulogiannis, 2018). The thrombin/PAR-1 interaction has been shown to activate the PI3K/Akt signaling pathway and induce IL-6 production at the fracture site in mouse femoral bones (Sato et al., 2016). Previous studies demonstrated that a PAR-1 antagonist attenuated myocardial and renal IR injury by inhibiting thrombin-induced activation of PI3K/Akt signaling (Strande et al., 2007; El Eter and Aldrees, 2012) Consistent with previous studies, the present study results showed that SCH530348 alleviated the activation of PI3K/Akt signaling and protected against IR injury in the lung. NF-κB has a pivotal role in mediating inflammatory responses by regulating the expression of proinflammatory mediators, including cytokines, chemokines, and adhesion molecules (Bakheet et al., 2020). NF-κB signaling is activated by phosphorylated IκBα, and free NF-κB translocates into the nucleus to initiate the transcription of inflammatory genes. Jeffers et al. (2015) demonstrated that through PAR-1, thrombin activates PI3K/Akt and NF-kB signaling to initiate an uncontrolled thromboinflammatory response in pleural mesothelial cells. In addition, a PAR-1 antagonist was shown to significantly ameliorate LPS-induced nuclear translocation of NF-κB and the degradation of IκB in microglial cells (Li et al., 2016). The results of the current study indicate that PAR-1 blockade suppresses the lung inflammatory response by attenuating NF-κB activation.

The MAPK signaling pathways, including p38, ERK1/2 and JNK, regulate inflammation and immune responses by modulating proinflammatory factors such as TNF-α and IL-6 (Nailwal and Doshi, 2021). TNF-α plays a critical role in the pathogenesis of inflammatory disease, and inhibition of TNF-α production has immunomodulatory and therapeutic effects (Ansari et al., 2021). Our previous study showed that the activation of MAPK pathways and TNF-α production were associated with IR-induced ALI (Liao et al., 2017). In addition, PAR-1 activation induces ERK phosphorylation in alveolar epithelial cells and increases lung inflammation during H1N1 infection (Khoufache et al., 2013). Furthermore, Wang et al. (2012) showed that the MAPK pathway was markedly activated after cerebral ischemia, and this upregulation was absent in PAR-1-knockout mice. Based on these findings and ours, a PAR-1 antagonist appears to protect against lung IR injury by attenuating MAPK signaling pathway activation. Notably, complex networks are involved in the crosstalk among the NF-κB, PI3K and MAPK signaling pathways. The NF-κB signaling pathway has been reported to be downstream of PI3K, and PI3K signaling blockade could suppress thrombin-mediated NF-κB activation (Jeffers et al., 2015). Previous studies have shown that the ERK signaling pathway is regulated by PI3K/Akt in global cerebral IR injury in rabbits and in human oral keratinocytes (Rohani et al., 2010; Yang et al., 2017). Another study also indicated that PI3K/Akt signaling was probably upstream of MAPK signaling in thrombin-activated osteoblasts (Sato et al., 2016). Based on the abovementioned findings, we speculated that PAR-1 activation might initiate PI3K/Akt signaling and phosphorylate downstream MAPK and NF-κB targets, thereby amplifying the inflammatory response in lung IR injury. In the current study, SCH530348 blocked PAR-1 signaling and thrombin/PAR-1 interaction during lung IR injury, subsequently suppressed PI3K/Akt, MAPK, and NF-κB signaling activation, prevented neutrophil recruitment, and decreased downstream proinflammatory cytokine production, including TNF-α, IL-6 and CINC-1. These results suggest that SCH530348 ameliorates lung IR injury by inhibiting PAR-1 activation and multiple inflammatory downstream signaling pathways.

There are some limitations to our study. First, we only found links between PAR-1 and the PI3K, NF-κB or MAPK pathways. However, we did not use specific inhibitors to show direct relationships between these pathways. Future studies specifically designed to answer this question will be needed. Second, we used only MLE-12 cells in this study. Further studies should be performed using other cell lines, such as endothelial cells, to clarify the mechanism of PAR-1-regulated lung IR injury.

In conclusion, the lung-protective effects observed in the current study appear to be related to the inhibition of PAR-1. A PAR-1 antagonist may have additional therapeutic utility in the treatment of IR-induced ALI.

## Data Availability

The original contributions presented in the study are included in the article/Supplementary Material, further inquiries can be directed to the corresponding author.
